# Spastic Constipation

**Published:** 1906-04-07

**Authors:** 


### SPASTIC CONSTIPATION.

This, according to Dr. Dudley Roberts,[1] is a common variety of chronic constipation but often escapes notice. It is described as being dependent upon a spastic contracture of a part or the whole of the lower bowel. Very often it is developed upon atonic constipation of long standing, but it may be primary. The condition was first described by Cherchewski in 1883. According to Albu, the frequency of its occurrence is so high as to make it responsible for no fewer than 25 per cent. of all cases of habitual constipation. The author considers even this proportion too low in the case of constipation among neurotic females.

This condition is entirely unbenefited by the ordinary measures taken to relieve sluggishness of the bowels. It is usually dependent upon neurasthenia, hypochondria, or hysteria, and is merely an evidence of irritable weakness. Occasionally a similar malady is met with among strong and phlegmatic men; but in these cases there is usually a history of a chronic tendency to constipation, which has led to the prolonged use of active cathartics.

The subjective symptoms of spastic constipation are suggestive without being characteristic. The usual complaint is that the ordinary cathartics are not satisfactory: that unless the stools are quite watery they are only passed with great difficulty; such patients will often recognise that the bowel is functionless in their attempts at defæcation, and that the scanty success which attends their efforts is due in the main to the expulsive action of the voluntary muscles, the diaphragm and the muscles of the anterior abdominal wall.

The character of the stools greatly assists the diagnosis, for in this condition the dejections are commonly of very small calibre—approaching that of a lead pencil. Palpation of the belly will sometimes reveal the spastic state of the colon, which may be recognised and rolled under the fingers as a tube of approximately the diameter of the index-finger. It may be possible to demonstrate that a part of the colon is distended by gas, while an immediately adjoining portion is in a state of spasm and rigidity. In cases exhibiting spasm of the lowest segment of the large intestine the spasm can be readily determined by rectal examination.

The prognosis is usually good, for even if the nervous abnormality cannot be cured, much may be done for its mitigation. The treatment must, in the first place, be directed towards the relief of any underlying neurasthenia or hypochondria. This involves careful attention to the diet, for a great preponderance of these patients are chronically underfed. In the second place moral treatment is of the highest value. "Many individuals form a habit of worrying about the action of the bowels, and in some this reaches a point where defæcation is actually inhibited. There is no doubt that some of these spastic conditions are induced by worries and fears. Much can be done with these individuals by a course of psychic treatments allied to suggestion."

In addition to the above, local treatment of the bowel is essential. The object of such treatment must be the relief of irritation, and the lessening of irritability, and in both departments diet is of the first importance. These patients have usually been in the habit of ingesting large quantities of fruit and coarse vegetables, on account of the admitted virtue of these elements of diet in relieving atonic conditions of the gut. Of course, when the condition at work is the opposite of atony, such a proceeding merely intensifies the evil. The diet should therefore be of the blandest and least irritating description. If fruits and vegetables are given at all they must be first passed through a sieve. Fats and sugars are usually well borne and conducive to laxity of the bowels. The tonic variety of hydrotherapeutics must be replaced by the sedative: that is to say cold douches and friction baths must be replaced by warm sitz baths and warm applications to the abdomen. All cathartics must be discarded in favour of clysters of oil, and particularly olive oil. These clysters are best given at night when the patient retires to bed; under these circumstances they are often retained for upwards of twelve hours. But it is well at first to apply a pad to the perineum after the injection, in case any leakage should take place. The quantity of oil injected will depend upon the site of the spasm, say from five to sixteen ounces, according to the age and size of the patient and the distance of the spastic area above the anus. Irritation of the bowel and consequent speedy expulsion of the clyster is less likely to occur if the oil before injection is warmed to a degree slightly above the body heat.

These injections should be given nightly for a week, the frequency being subsequently diminished until, after an interval of three or four weeks, it may be found possible to discontinue them entirely. In addition to the oil-injections, suppositories containing hyoscyamus or belladonna have a definite value; while, as a further antispasmodic, it may be well to administer bromides for a short space of time. The cure may be a matter of weeks or months according to the degree of nervous irritability encountered.

[1] Brooklyn Med. Jour., March, 1906.

